# Evidence for intracellular *Pseudomonas aeruginosa*

**DOI:** 10.1128/jb.00109-24

**Published:** 2024-04-10

**Authors:** Zachary J. Resko, Rachel F. Suhi, Adam V. Thota, Abby R. Kroken

**Affiliations:** 1 Department of Microbiology and Immunology, Loyola University Chicago, Maywood, Illinois, USA; Geisel School of Medicine at Dartmouth, Hanover, New Hampshire, USA

**Keywords:** *Pseudomonas aeruginosa*, facultatively intracellular pathogens, secretion systems, host–pathogen interactions, intracellular pathogen, host cell invasion, epithelial cells

## Abstract

*Pseudomonas aeruginosa* is a significant cause of global morbidity and mortality. Although it is often regarded as an extracellular pathogen toward human cells, numerous investigations report its ability to survive and replicate within host cells, and additional studies demonstrate specific mechanisms enabling it to adopt an intracellular lifestyle. This ability of *P. aeruginosa* remains less well-investigated than that of other intracellular bacteria, although it is currently gaining attention. If intracellular bacteria are not killed after entering host cells, they may instead receive protection from immune recognition and experience reduced exposure to antibiotic therapy, among additional potential advantages shared with other facultative intracellular pathogens. For this review, we compiled studies that observe intracellular *P. aeruginosa* across strains, cell types, and experimental systems *in vitro*, as well as contextualize these findings with the few studies that report similar observations *in vivo*. We also seek to address key findings that drove the perception that *P. aeruginosa* remains extracellular in order to reconcile what is currently understood about intracellular pathogenesis and highlight open questions regarding its contribution to disease.

## INTRODUCTION

*Pseudomonas aeruginosa* is a ubiquitous Gram-negative opportunistic pathogen that is the causative agent of numerous severe human health conditions in both immunocompetent and immunocompromised individuals. It is among the five leading pathogens accounting for more than half of global bacterial-related deaths (1). Significant clinical manifestations include skin and soft tissue infections, pneumonia, urinary tract infection, ulcerative keratitis, and sepsis. *P. aeruginosa* was initially regarded as an extracellular pathogen based on observations where adherence to epithelial cells of mucosal surfaces preceded toxin-mediated inflammation, immune cell recruitment, and tissue damage (2–4). The discovery of its type three secretion system (T3SS) (5) and anti-internalization activities of T3SS effectors (6) fueled the perception that it operates exclusively as an extracellular pathogen. Nevertheless, just over 100 studies have observed *P. aeruginosa* persisting or even thriving within host cells, many of which investigate mechanisms enabling this phenomenon. In this review, we examine the contributions of those studies to our current understanding of *P. aeruginosa* pathogenesis and seek to reconcile disparate observations of where bacteria go within hosts, highlight factors enabling them to inhabit host cells, and consider how bacteria might contribute to disease from intracellular locations.

## EARLY INVESTIGATIONS OF *P. AERUGINOSA* INTERNALIZATION INTO CELLS

*P. aeruginosa* produces many secreted virulence factors, including proteases such as elastase B and alkaline protease (7), and Exotoxin A, a mono-ADP-ribosyltransferase (ADPr) targeting elongation factor 2 (8). Due to secreted products that damage host cells and digest extracellular matrix proteins, combined with a lack of explicit evidence that *P. aeruginosa* entered cells similarly to other well-studied facultatively intracellular bacteria, it was generally viewed as an extracellular pathogen. Early studies to identify mechanisms of *P. aeruginosa* virulence focused on characterizing the determinants of epithelial cell adherence and cytotoxicity, as well as secreted proteins that damage cell-surface and extracellular matrix proteins. These established the importance of pili and flagella for adherence, and Exotoxin S (ExoS) for tissue penetration and dissemination even before the discovery of a T3SS in *P. aeruginosa*, which injects ExoS into target cells (9, 10).

The earliest records of intracellular *P. aeruginosa* are primarily observational snapshots using transmission electron microscopy (TEM), or experiments that count colony forming units (CFU) after killing extracellular bacteria with an antibiotic that does not penetrate host cell membranes. One of the first is a report from 1985 showing internalization of *P. aeruginosa* into epithelial cells of abraded New Zealand white rabbit corneas (11). A study performed in 1990 using a rabbit contact lens model also observed epithelial cells with intracellular bacteria (12). In 1987, a report remarking upon an “unusual” pathogenic mechanism showed a cystic fibrosis (CF) isolate growing within polymorphonuclear neutrophils (PMNs), whereas other strains of *P. aeruginosa* were killed by PMNs (13). Moving into tissue culture experiments, a 1991 study examining extracellular interactions between *P. aeruginosa* strain PAK and pneumocytes showed that a small proportion of lung epithelial cells harbored intracellular *P. aeruginosa* within endocytic vesicles (14). In the years following, intracellular *P. aeruginosa* was observed by microscopy in additional epithelial cell types including tracheal, corneal, and intestinal, revealing bacteria favored association and invasion of cells within damaged tissues (15–17). Experiments using mice observed intracellular bacteria in neonatal lung epithelial cells (18) and corneas (16). Thus, like several other pathogens that exhibit an intracellular stage in their life cycle, *P. aeruginosa* is also capable of surviving in intracellular locations, although early observations differed in which subcellular location bacteria were found.

Although evidence suggested that *P. aeruginosa* is capable of epithelial cell invasion, relatively little was known about the relevance of these observations to the overall pathogenicity and survival of this bacterium. A series of reports beginning in the mid-1990s (many contributed from the laboratory of Gerald B Pier at Harvard Medical School) began to dissect the basis for *P. aeruginosa* invasion within epithelial cells *in vivo* and *in vitro,* focusing on two relevant sites: corneal epithelium, where *P. aeruginosa* is a significant threat to vision following injury or contact lens wear, and respiratory epithelium with regard to the cystic fibrosis transmembrane conductance receptor (CFTR), which is defective or absent in cystic fibrosis patients, rendering them susceptible to chronic *P. aeruginosa* respiratory infections.

One of the first reports to quantify intracellular presence and replication of bacteria used a mouse corneal injury model. Bacterial invasion and survival within corneal cells were quantified over a 24-hour period. Invasion by *P. aeruginosa* strain 6294 (a corneal infection isolate) was detected as early as 15 minutes post-infection and increased steadily over time, where approximately 22% of the recovered CFU was intracellular as determined by a gentamicin protection assay (16). TEM showed *P. aeruginosa* within membrane-bound vacuoles at 8 hours post-infection, suggesting that uptake occurred through an endocytic process (16). By 24 hours post-infection, bacteria were detected in the cytoplasm of infected corneal cells, indicating escape from the endocytic vacuole (16). Compared to the invasion rate observed in injury infection models and whole corneas *in situ*, primary corneal cells isolated from animals or humans were invaded more efficiently by *P. aeruginosa in vitro* (19). Hence, primary corneal cells in culture were used for mechanistic investigation of the processes involved in *P. aeruginosa* entry, survival, and replication. Infected primary corneal cells showed a 10-fold increase in intracellular CFU burden between 1 and 4 hours post-infection, implying that *P. aeruginosa* is capable of intracellular multiplication shortly after invasion; CFU quantity decreased back to input levels by 24 hours likely due to epithelial cell death (19). In the cornea, internalization into epithelial cells was predicted to be advantageous for bacterial colonization, and potentially contributes to disease severity and ulcers that remain difficult to treat.

Considering the susceptibility of cystic fibrosis patients to *P. aeruginosa* lung infections, several studies investigated the role of CFTR, which led to the first mechanistic investigations of bacterial internalization into lung epithelial cells. Here, the data supported a hypothesis that bacterial “ingestion” was host-driven and beneficial for clearing *P. aeruginosa* from airways (20). Pier and colleagues first confirmed that lung epithelial cells could internalize *P. aeruginosa* bacteria in 1996 (21). In this context, CFTR acts as a specific receptor for *P. aeruginosa* internalization: CFTR with the ∆F508 mutation associated with cystic fibrosis is retained in the endoplasmic reticulum and ultimately degraded (22, 23), leading to decreased numbers of bacteria internalized (24). When CFTR ∆F508 is forced to the surface, bacterial uptake is restored (21). The first extracellular domain of CFTR was shown to bind complete-core lipopolysaccharide from the outer membrane of *P. aeruginosa* (24), and bacterial invasion could be inhibited by blocking CFTR amino acids 108–117 with a monoclonal antibody (25). However, artificial overexpression of CFTR decreased the probability of *P. aeruginosa* internalization (26). This may occur because *P. aeruginosa* internalization also requires free lateral diffusion of both CFTR and caveolin-1 within a distinct subset of lipid rafts (27), which may be impacted by artificial overexpression. Therefore, optimal composition of CFTR-containing lipid rafts appears important for maximum uptake of bacteria. Taken together, the data showing CFTR-mediated internalization of *P. aeruginosa* into lung epithelial cells led to a model suggesting epithelial cells promote bacterial clearance in airways, which would be impaired in cystic fibrosis patients (20, 28). A role for CFTR has also been investigated in ocular surface cells, showing that hypoxic conditions increased levels of CFTR expression in rabbit corneas and cultured corneal epithelial cells, which correlated to increased *P. aeruginosa* internalization in these models (29).

However, other studies put forth the idea that reduced CFTR-mediated clearance does not fully explain susceptibility to chronic infections in cystic fibrosis patients, or they were unable to verify clearance even occurred proportionately to mouse or human CFTR expression in animals (30). Other work showed that internalization was driven by polarity of the host cells tested, which *correlated* with CFTR localization, although CFTR itself was dispensable in this system (31). Another study showed ∆F508 CFTR-expressing cells internalized more *P. aeruginosa* than cells expressing wild-type (WT) CFTR (32). A latter study delineating internalization rate from intracellular multiplication of bacteria found that although internalization was reduced in CFTR mutant cells, intracellular multiplication of bacteria was enhanced; as such, the exact timing of bacterial enumeration could influence findings (33).

The precise role of CFTR on *P. aeruginosa* internalization remains complex, as does the contribution of intracellular bacteria during progression of infection in cystic fibrosis patients. Its impact may be modulated by host cell membrane composition, or it may promote internalization only into specific types of epithelial cells.

## BACTERIAL INTERNALIZATION MECHANISMS INTO NON-PHAGOCYTIC CELLS

Some intracellular pathogens use T3SS effectors to promote invasion by manipulating the host cytoskeleton, such as SopE in *Salmonella* (34) or VirA in *Shigella* (35). Because *P. aeruginosa* lacks an apparent effector that induces uptake, other host and bacterial factors that promote or facilitate internalization have been investigated.

To identify host factors required for *P. aeruginosa* uptake, early studies used inhibitors of host processes. Use of cytoskeletal polymerization inhibitors corroborated the requirement for a host endocytic process for invasion, as treatment of corneal cells with cytochalasin D prior to infection with *P. aeruginosa* abrogated internalization (19, 36). Invasion could also be reduced using tyrosine kinase inhibitors (37, 38). Mutation of the C-terminal Src kinase (Csk), a regulator of Src tyrosine kinases, also reduced invasion of fibroblasts, further suggesting an important role for actin cytoskeleton remodeling in *P. aeruginosa* internalization (39). The MEK-ERK signaling module, one of three known mitogen-activated protein kinase pathways, has also been associated with bacterial internalization (40).

Based on the proclivity of *P. aeruginosa* to invade damaged areas of epithelial cells, cellular junction integrity and membrane polarity were also explored as possible host factors that prevent bacterial invasion. Disruption of tight junctions and exposure of the basolateral membrane via EGTA treatment rendered Madin-Darby canine kidney (MDCK) cells, human corneal epithelium, and human nasal epithelium more susceptible to *P. aeruginosa* invasion (41). Bloodstream infection isolates were also found to penetrate Caco-2 cell monolayers better than isolates from sputum, which was attributed to their increased ability to disrupt cellular junctions and high production of Exotoxin A (42). These observations shaped the hypothesis that epithelial cell receptors involved in *P. aeruginosa* internalization may be localized to the basolateral membrane in polarized epithelial cells, and that invasion occurs preferentially at basolateral surfaces, which are exposed by damage. This would be similar to preferential invasion of *Shigella* at basolateral surfaces of intestinal epithelial cells (43).

*P. aeruginosa* internalization can also be influenced by host membrane lipid composition. Bacteria have been shown to interact with lipid rafts on the outer leaflet of the host cell plasma membrane. In a rabbit model of contact lens wear, Yamamoto et al. found that corneal cells with exposed lipid rafts preferentially bound and internalized *P. aeruginosa* (44). When glycosphingolipids are depleted, *P. aeruginosa* binding and internalization was inhibited (45). In a follow-up study, they demonstrated that conjunctival epithelial cells possessing lipid rafts and CFTR failed to bind *P. aeruginosa*, suggesting CFTR may not function as a receptor for *P. aeruginosa* in the conjunctiva (46). Interactions between the glycosphingolipid asialo-GM1 and Type IV pili have been shown to mediate adherence and invasion of epithelial cells (47). Another study found a role for phosphatidylinositol (3,4,5)-triphosphate (PIP_3_) in the binding and internalization of *P. aeruginosa* aggregates in MDCK cells through the function of another Src family tyrosine kinase, Lyn (48).

Further studies into the importance of lipid composition led to a proposed lipid zipper model, which was facilitated through bacterial surface lectins. Eierhoff et al. discovered a bacterial surface lectin, LecA, which interacts with the glycosphingolipid Gb3 allowing uptake of *P. aeruginosa* (49). In a human lung epithelial cell line, LecA–Gb3 interaction was shown to induce the clustering of PIP_3_, flotillins, and CD59 in the plasma membrane at sites of LecA binding and promote uptake. Depletion of these host cell components reduced bacterial invasion (50). Aigal et al. reported formation of septin barriers at LecA binding sites, which may inhibit *P. aeruginosa* uptake by increasing local membrane rigidity, adding a caveat to this model (51).

Lectins also influence bacterial access to basolateral membranes of host cells (52). The *P. aeruginosa* surface lectin, LecB, was shown to bind *β*1-integins causing rapid integrin endocytosis, which loosened cell-substrate adhesion, permitting *P. aeruginosa* to “crawl” underneath polarized cells (53). LecB was also shown to bind to fucosylated receptors at the apical plasma membrane of epithelial cells, which results in a Src-PI3K/Akt signaling cascade ultimately leading to the recruitment of caveolin-1 to the apical domain and enhanced invasion efficiency of *P. aeruginosa* (54).

Other bacterial surface factors that are predicted to influence surface attachment and subsequent invasion include the flagella, which is thought to facilitate cellular association through binding of N-glycans of polarized epithelial cells (55, 56). An additional mechanism promoting *P. aeruginosa* internalization is the H2 type VI secretion system (H2-T6SS), which was shown to enhance *P. aeruginosa* uptake into HeLa cells (57). In subsequent work, the H2-T6SS effector VgrG2b was shown to promote microtubule-dependent uptake of *P. aeruginosa* through an interaction with the γ-tubulin ring complex, which is a eukaryotic multiprotein complex that facilitates microtubule nucleation (58). Evidence also suggests that the constitutively expressed multidrug efflux system, MexAB-OprM, is important for invasion and traversal of epithelial cell layers. Although an exact mechanism has yet to be identified, it is postulated that the efflux system contributes to export of virulence factors along with other harmful compounds (59).

**Fig 1:**
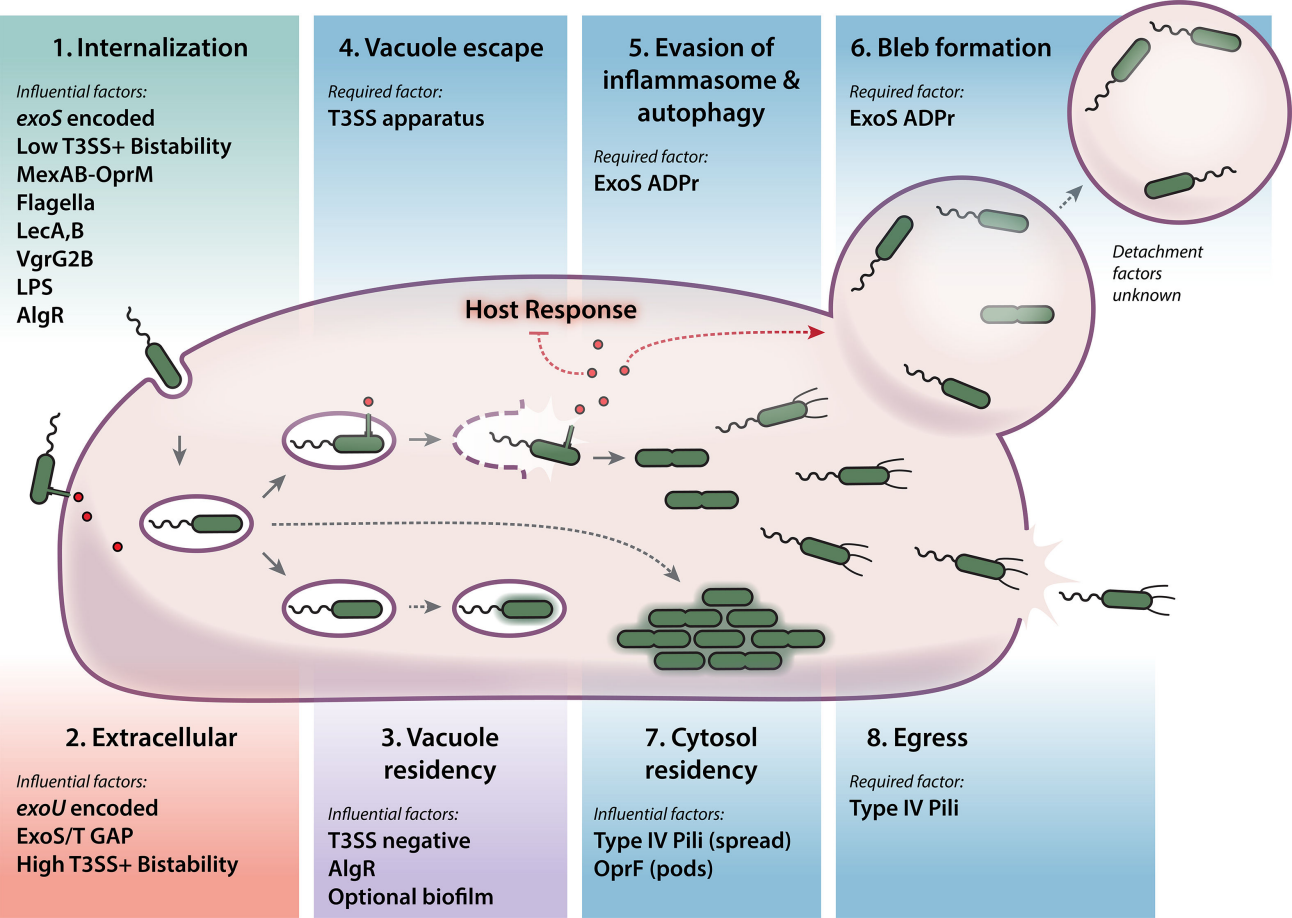
Known factors that influence *P. aeruginosa* invasion and intracellular trafficking. Factors influencing *P. aeruginosa* to invade epithelial cells are listed in panel 1, *Internalization* (top left). Alternatively, factors that influence bacteria to remain extracellular are shown in panel 2, *Extracellular* (bottom left). Bacterial factors promoting survival within vacuoles are shown in panel 3, *Vacuole residency*. Should bacteria exit vacuoles, the subsequent elements influencing the trafficking of bacteria into the cytoplasm, and events such as spread, membrane bleb entry, intraepithelial pod formation, and egress, are shown in panels 4–8. Solid arrows differentiate steps where mechanisms have been identified. Dashed arrows indicate observations where mechanisms remain to be elucidated or investigated.

A list of known bacterial factors that can promote internalization is provided in Figure 1 (see panel 1). Considering that each of these mechanisms was discovered in different experimental systems, some mechanisms may dominate in some host tissues, or a combination may influence the probability of invasion into specific cell types.

## DUAL FUNCTIONALITY OF TYPE THREE SECRETION SYSTEM EFFECTORS

Coincident with early mechanistic studies on bacterial internalization in the mid-1990s, discovery of a T3SS encoded by *P. aeruginosa* and the functions of its exoenzymes advanced the hypothesis that *P. aeruginosa* remains extracellular. Yahr et al. (5) showed in 1996 that ExoS, appreciated at the time as an ADPr enzyme distinct from Exotoxin A (60), was secreted through a T3SS . Following this came the discovery of three other T3SS exoenzymes, Exotoxin T (61), Exotoxin Y (62), and Exotoxin U (63). Around the same time as the discovery of the T3SS, clinical *P. aeruginosa* strains were typified as invasive (ExoS producing) or cytotoxic (ExoU producing) (64) on the basis that invasive strains could survive gentamicin protection assays and replicate (65), while cytotoxic strains rapidly lysed mammalian cells (66) (Fig. 1, see panels 1 and 2). However, as each exoenzyme’s mechanism of action was identified, the paradigm of invasive *exoS*-encoding strains began to shift.

ExoT is ubiquitously encoded among T3SS-positive *P. aeruginosa* strains (67) and consists of an N-terminal GTPase-activating protein (GAP) domain (68) and a C-terminal mono-ADPr domain (69). ExoS is only encoded in a subset of strains (67). It has identical domain architecture to ExoT (70) and shares ~76% amino acid identity (71). Although both ExoS and ExoT require a eukaryotic cofactor, a 14-3-3 protein, for ADPr activity, their target host substrate specificity differs (72, 73). ExoT ADP-ribosylates host targets Crk1 and Crk2, which are components of focal adhesions (74). ExoS ADPr has promiscuous substrate specificity primarily directed toward several families of small GTPases and the Ezrin/Radixin/Moesin (ERM) protein family (reviewed by Barbieri and Sun [71]). Data support the notion that additional substrates of ExoS remain to be identified (75). ExoY is a nucleotidyl cyclase (62) requiring actin as a cofactor (76, 77) and is expressed in a subset of both cytotoxic and invasive strains. ExoU is a potent A2 phospholipase (78) requiring ubiquitin as a cofactor (79) and is thought to rupture host cell membranes shortly after delivery.

In contrast to early observations of *exoS*-encoding strains invading host cells, ExoS itself was shown to block phagocytosis shortly after the discovery of its secretory mechanism. Phagocytic blockage was first demonstrated in a study that expressed ExoS in *Yersinia pseudotuberculosis*, in which the cell contact-mediated secretion of T3SS effectors was better understood at the time (80). Here, the N-terminus, (later identified as a RhoGAP domain [70]) provided an anti-phagocytic effect toward macrophages, as catalytic mutants of the ExoS ADPr domain remained capable of inhibiting phagocytosis of bacteria. The GAP domain of ExoT was the first shown to limit uptake of *P. aeruginosa* bacteria (6, 68); it followed that the biochemically similar GAP domain of ExoS could do the same. ExoY was also shown to have an inhibitory effect on bacterial invasion due to actin disruption, although this phenotype was only observable early in infection (81). Anti-internalization properties of ExoS GAP were also validated in corneal epithelial cells (6) and HeLa cells (82). Further, HeLa cells pre-intoxicated with ExoS prevented any subsequent invasion by WT and effector-null mutant *P. aeruginosa* (83). The ADPr domain of ExoS can also block phagocytosis of lung leukocytes, with a limited contribution of the GAP domain (84). It is generally thought that bacteria *activate* RhoGTPases using RhoGEF activity to enhance internalization (e.g., SopE-like effectors and the WxxxE effectors [85]), while bacterial RhoGAPs such as YopE from *Y. pseudotuberculosis* limit phagocytosis by disrupting actin polymerization (86, 87), or in the case of *Salmonella* SptP, promote restoration of RhoGTPase function after transient SopE activity (88). Thus, it was reasonable to postulate that RhoGAP activity of ExoS and ExoT served to keep bacteria from being phagocytosed.

How can the anti-phagocytic properties of ExoS and ExoT be reconciled with the ability of *exoS*-encoding strains to invade cells and replicate? We propose three possibilities.

First, the cell type may influence both T3SS activation and outcomes for internalized *P. aeruginosa*. Immune cells are discussed in more detail later; however, utilization of cytoplasmic niches has only been observed in epithelial cells. Otherwise, bacterial internalization into macrophages or neutrophils appears to lead to either death of the bacterium or host cell death without intracellular bacterial replication (74). Further, both T3 effector quantity (89), target specificity (90), and host cell responses (91) to ExoS vary by cell type. Thus, the relative ability of ExoS (and ExoT, by extension) to block bacterial uptake may vary according to cell type; epithelial cells may be more likely to support live intracellular bacteria than immune cells.

Second, one of the many substrates of ExoS is its own GAP domain catalytic residue, R146. Because the ADPr domain requires a host 14-3-3 cofactor, inactivation occurs after ExoS is secreted into a host cell. The kinetics of this modification, and how bacterial uptake could be modulated by transient GAP activity, remains to be investigated. ExoS expression can indeed reduce invasion into both epithelial (6) and phagocytic cells (80), so the GAP activity of ExoS is not completely nullified. Whether the GAP domain of ExoT is a target of ExoS is also an open question (71). If this is the case, then ExoS ADPr could regulate the function of both its own GAP domain and that of ExoT. Although this is an appealing explanation as to how invasive *P. aeruginosa* strains could have evolved through gene duplication of *exoT* and evolution of expanded ADPr substrate specificity (71), one experiment that posed this question failed to find evidence that ExoT is ADP-ribosylated by ExoS (92). Other explanations for ExoS overriding the anti-internalization ability of ExoT may have to do with secretion feedback inhibition by ExoS, which limits the total quantity of exoenzymes secreted by bacterial populations in contact with host cells (89, 93).

Third, experimental observations of anti-phagocytotic activity may be overrepresented in studies that used strain PA103. PA103 was useful as an early resource for both exotoxin A (owing to its low production of degradative proteases [8, 94]) and T3SS studies (38, 95). It is a cytotoxic strain encoding *exoU* and *exoT* and is more amenable to electroporation compared to other laboratory strains and clinical isolates. A strain of PA103 containing insertional mutations in *exoU* and *exoT* was used as an efficient tool to deliver plasmid-encoded ExoS to cell lines (70). PA103 also has some unusual properties, including missing flagella (96) due to a correctable point mutation in *fleQ* (97), and a high probability for most of the bacterial population to become T3-positive when activated by calcium chelation or host cell contact, which is not exhibited by other lab strains (83, 98). Heterogenous expression of the T3SS has been termed bistability (98), with a high or low “setpoint” indicating the proportion that become T3-positive upon triggering. Restoration of flagellar motility enhanced invasion of PA103∆*exoUT* into nonpolarized HeLa cells; however, the high bistable T3SS setpoint of PA103 correlated with a lower invasion rate when ExoS was provided by plasmid (83).

**Fig 2:**
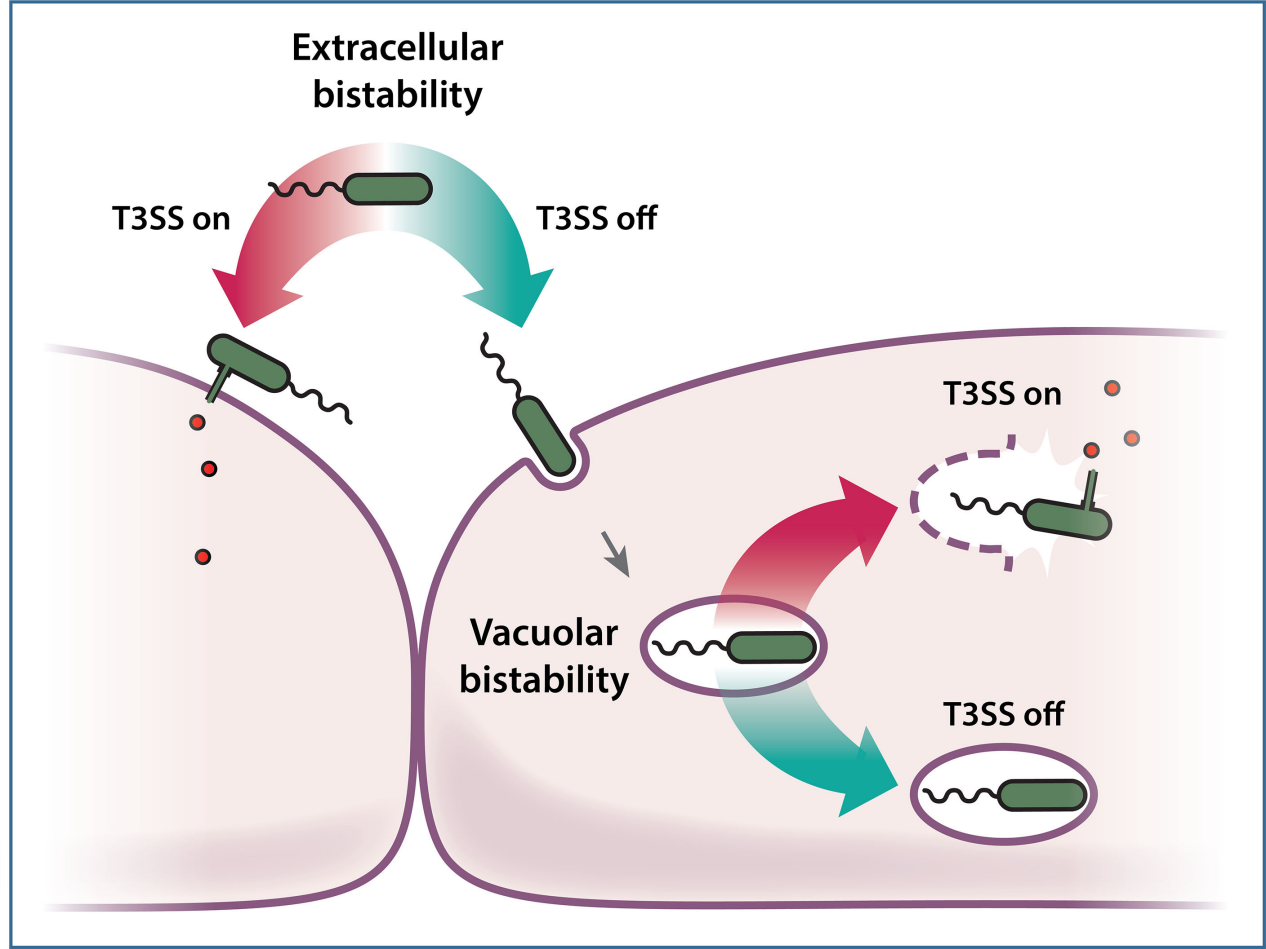
Bistability of T3SS expression may influence the location of *P. aeruginosa*. The T3SS can be induced by host cell contact, or *in vitro* by using calcium chelation. Only a sub-population of bacteria express the T3SS upon stimulation, a phenomenon termed bistability. Some bacteria remain T3SS negative upon host cell contact and are more likely to be internalized. These bacteria may instead express the T3SS after internalization, which is correlated with vacuole exit and entering host cell cytoplasm.

We envision a model in which conditions favoring a low bistability setpoint, where a large majority of the bacterial population is T3SS negative, leads to increased bacterial internalization (Fig. 2). In support of this model, a significant population of internalized bacterial cells do not appear to become T3SS positive and remain in vacuoles (99). Factors influencing bistability in vacuolar environments may be different to extracellular spaces (e.g., quorum sensing molecule concentrations, which are known to repress the T3SS [100]).

## DISTINCT BACTERIAL POPULATIONS OCCUPY SPECIFIC NICHES IN EPITHELIAL CELLS

Invasion and replication of *P. aeruginosa* have been shown in numerous epithelial cell types including corneal epithelial (16), MDCK (41), HeLa (101), bronchial epithelial (33), and urinary epithelial cells (102). Beyond gentamicin protection assays to measure intracellular CFU at varied times, several studies have followed up on key questions: (i) Where do bacteria reside within cells? (ii) Which virulence factors enable replication? (iii) What host cell defenses are engaged and how are they mitigated? Figure 1 (see panels 3–8) depicts the current known sequence of events and optional points of divergence. Factors influencing each step are discussed in detail below.

Early efforts to probe the subcellular location of invasive *P. aeruginosa* yielded mixed findings. A striking observation made using phase-contrast imaging showed bacteria exhibiting swimming motility within membrane blebs protruding from corneal (103) and bronchial epithelial cells (33), and HeLa cells (83). Blebs typically ranged from 10 to 35 µm in diameter. Because the blebs were not amenable to formaldehyde fixation, studies of their formation and properties were limited to techniques compatible with live cell imaging (33). Blebs formed independently of actin contraction, appeared mechanistically distinct from apoptotic blebs, and could be collapsed and inflated by manipulating osmolarity (33).

Generation of membrane blebs and bacterial occupation of them are both dependent on ExoS ADPr activity (104). Bacteria lacking ExoS, or the entire T3SS, were instead occasionally observed tumbling inside moderately sized vacuoles (103, 104). Correlating with this observation, gentamicin protection assays yield limited or reduced CFU recovered over time when ExoS ADPr or the entire T3SS was mutated (104). Some evidence suggests that ExoY may also facilitate similar large bleb formation but does not promote intracellular replication like ExoS (105, 106). Phase-contrast imaging limits reliable detection of bacteria to translucent spaces such as vacuoles or blebs; use of fluorescent bacteria in addition to TEM revealed that both wild-type and ExoS-null strains can enter the cytoplasm of host cells (83, 92, 99) and migrate throughout the cell using Type IV pili-mediated motility (107). Cytosolic entry, motility, and some replication occur prior to bleb formation, which typically occurs 7–12 hours post infection (83). Suppression of bleb volume using osmotic manipulation limits bacterial multiplication (33). Not all cytoplasmic bacteria enter the bleb as it forms, and ExoS delivery into host cells from extracellular bacteria is also sufficient to induce empty bleb formation (83). Blebs may also detach and continue to protect occupant bacteria, which may be a potential source of dissemination (108). Alternatively, cellular exit has also been attributed to twitching motility (109). Thus, it remains unclear if the bleb itself is always advantageous or merely coincides with other host processes impacted by ExoS.

Vacuolar bacteria initially visualized by phase-contrast imaging may be accounted for by a population that remains T3SS negative (99, 110). Mutants of the T3SS translocon (e.g., *popB*) are also confined to vacuoles (110), thus entry into the cytosol requires the entire T3SS apparatus but does not require the secreted effector toxins. This is similar to what has been demonstrated when the *Shigella* T3SS is expressed in a heterologous system: the T3 apparatus is sufficient to exit vacuoles (111). Whether vacuolar escape is mediated by other proteins secreted through the T3SS (112, 113) has not yet been tested. A recent study identified that a subset of T3SS-negative vacuolar bacteria can upregulate biofilm-related genes such as *cdrA* (99), raising questions about when populations displaying chronic infection characteristics arise during acute infection. Mixed populations of T3SS-positive cytoplasmic bacteria and T3SS-negative vacuolar bacteria could account for varied reports of where bacteria reside within different types of cells. It is conceivable that the cell type influences which outcome is more favorable.

Regarding intracellular bacteria upregulating biofilm production, an earlier study identified biofilm-like aggregates within primary mouse tracheal epithelial cells grown on air–liquid interface trans-well dishes (114). Here, bacteria formed large intracellular aggregates that resisted killing by ceftazidime and ciprofloxacin while inside cells, although the bacterial aggregates could be made susceptible if disrupted mechanically. This report put forth a model that bacteria may be able to accumulate into intraepithelial “pods,” which leads to occupied epithelial cells being ejected from the epithelial monolayer (Fig. 1) (114). This finding also agreed with a prior study that sought to identify whether lung epithelial cells could support chronic infection and observed sloughing cells with bacterial aggregates (28). From there, bacteria within dead epithelial cells might be cleared by professional phagocytes, or alternatively, be retained in the airway in individuals with compromised immune systems.

A recent study on intracellular *P. aeruginosa* in bladder epithelial cells discovered stable intracellular populations (102). Here, *P. aeruginosa* exhibits a slow doubling time, as most cells harbored fewer than 10 intracellular bacteria, which were primarily found within LAMP-1-positive compartments. This study also used dual RNA-seq transcriptional profiling to identify bacterial adaptations to the intracellular host environment and lends further evidence to intracellular populations resisting antibiotic treatment. A mutant of a mucoid transcriptional regulator, *algR*, exhibited lower invasion frequency and reduced proliferation rate. This differs from the cytosolic T3SS-positive population found in corneal and airway epithelial cells, and supports the notion that there are different bacterial responses inside different types of host epithelial cells. Persistent intracellular populations of *P. aeruginosa* have also been observed in an airway epithelial cell line, BEAS-2B. After infection with either the lab strain PAO1 or CF isolates and continuous tobramycin treatment, airway epithelial cells harbored intracellular *P. aeruginosa* up to 120 hours post-infection (115).

Given this heterogeneity in the bacterial factors employed in establishing intracellular niches (Fig. 1) between different epithelial cell types, it is possible that *P. aeruginosa* utilization of intracellular niches may be more important (and more readily observable) in some infection sites over others.

## EVASION OF EPITHELIAL INTRACELLULAR HOST DEFENSE

Only some bacterial species have evolved the ability to grow within host cell cytoplasm while also evading host detection and response. Conversely, introducing non-intracellular pathogens into the cytoplasm by microinjection does not enable them to replicate there (116). Bacteria internalized into host cells are generally trafficked to lysosomes for killing. They avoid this fate in multiple ways: disrupting lysosomal trafficking and remodeling a specialized vacuole (e.g., *Salmonella*, *Chlamydia*), exiting the phagolysosome altogether for the cytosol (*Shigella*, *Listeria*), or surviving low pH (*Coxiella*). Considering where *P. aeruginosa* can reside, it likely has methods of surviving early endosomes (99), evading or neutralizing lysosomes (102, 110, 117), and surviving within the cytosol while evading host detection for a short time (92).

A study by Heimer et al. found that *P. aeruginosa* mutants lacking the T3SS were confined to acidified perinuclear vacuoles at a higher frequency than WT, which can occupy both membrane blebs and vacuoles. However, the vacuoles containing WT *P. aeruginosa* were significantly less acidified than T3SS mutant-bearing vacuoles (110). In accordance with this data, another study found that T3SS mutant *P. aeruginosa* associated with LAMP-3-positive vacuoles at a higher rate than WT, potentially suggesting that ExoS may prevent lysosomal fusion or vacuolar acidification (103). Potentially, this could occur by disrupting trafficking through ADP-ribosylation of Rab5 or other Rab proteins (82, 118). Currently, the mechanism underlying the role of ExoS ADPr activity in the prevention of lysosomal fusion to vacuole and redirection of *P. aeruginosa* to membrane blebs remains to be elucidated.

ExoS ADPr can attenuate other host cell responses. Recent work from Rao et al. demonstrated ExoS ADPr mediates autophagy inhibition on two independent fronts in airway epithelial cells (119). First, ExoS inhibits the mTOR pathway through ADP-ribosylation of host Ras GTPase, effectively inhibiting the Ras signaling pathway and downstream mTORC1 activity. Under normal circumstances, mTOR inhibition would activate autophagy and negatively impact intracellular pathogen survival. However, *P. aeruginosa* mitigates this activation of autophagy on a second front: ExoS ADPr activity leads to suppression of the III phosphatidylinositol 3-kinase Vps34, which plays a pivotal role in formation of early autophagosomes (119).

Pyroptosis is an inflammasome-mediated response to pathogen- or damage-associated molecular patterns (PAMPs or DAMPs) detected in host cell cytosol. *P. aeruginosa* can trigger several inflammasome pathways. This has largely been investigated in immune cells, generally not from the perspective of bacteria becoming cytosolic. For example, flagellar proteins may be injected to the cytosol through the T3 apparatus while bacteria remain extracellular to activate NLRC4 (120), or lipopolysaccharide can be delivered by outer membrane vesicles to activate caspase-11 (121). Recent work identified that a strain missing exotoxins but encoding the T3SS apparatus can enter the cytoplasm of corneal epithelial cells but causes rapid host cell death (99), which was identified as pyroptosis mediated by the caspase-4 inflammasome (122). Host cell survival and maintenance of a cytoplasmic niche for bacterial multiplication were dependent on ExoS ADPr activity (92), although the target-mediating suppression of pyroptosis remains to be identified. *P. aeruginosa* has also been reported to activate the non-canonical inflammasome in mouse macrophages, although only one study investigates whether bacteria became intracellular (121, 123).

Because cytoplasmic entry appears to be dependent on expression of the T3SS apparatus, but not ExoS, ExoT, or ExoY (83), the ability to access the cytoplasm and replicate within host cells appears to be conserved even in cytotoxic (ExoU-encoding) strains. This can be observed when ExoU and T are deleted (38, 83). Thus, any required adaptations to replicate in host cell cytoplasm are widely conserved in *P. aeruginosa* strains, suggesting survival in this space may be “accidental” and not an evolutionary response to specific host-selective pressures.

## ENCOUNTERS AND OUTCOMES WITH PHAGOCYTIC IMMUNE CELLS

Although *P. aeruginosa* can establish an intracellular replicative niche within numerous epithelial cell types, currently, no evidence suggests it can usurp phagocytic immune cells in a similar manner. Rather, immune cell recruitment, inflammatory cytokine production, and phagocytosis by neutrophils and macrophages contribute to bacterial clearance and infection resolution (124, 125). Accordingly, immunocompromised patients or those with conditions associated with immune dysfunction, such as chronic obstructive pulmonary disease, are hypersusceptible to *P. aeruginosa* infections, likely because bacterial numbers remain uncontrolled (126).

Although the ability of *P. aeruginosa* to exploit phagocytic immune cells for intracellular replication has yet to be definitively observed, some reports demonstrate various mechanisms of immune evasion that contribute to pathogenicity. It is generally thought that NLRP3 inflammasome activation following phagocytosis and subsequent immune cell autophagy promotes bacterial clearance. However, *P. aeruginosa-*induced autophagy in human macrophages reduces their phagocytic capacity and thereby suppresses intracellular bacterial killing (127). As previously mentioned, *P. aeruginosa* T3SS effectors ExoT and ExoS also contribute to immune evasion by preventing phagocytosis through disruption of actin cytoskeleton polymerization (68, 84). Further, ExoS has been shown to facilitate phagosomal escape of internalized bacteria, although entry into macrophage cytoplasm leads to immediate cell lysis, not intracellular niches for replication (74, 128). Despite the T3SS serving a similar function in both epithelial cells and phagocytes by aiding bacterial egress from phagocytic compartments, the differences between these host cell types in inflammatory signaling capacity seemingly dictate whether *P. aeruginosa* can establish a replicative niche or will initiate inflammatory cell death. This dichotomy may be attributed to the multiple pattern recognition receptors and inflammasome pathways activated upon bacterial recognition by phagocytes. *P. aeruginosa* activates both NLRC4 and NLRP3 inflammasome pathways in a T3SS-dependent manner (123). Intriguingly, in *caspase-11*^-/-^ bone marrow-derived macrophages (BMDMs), a *popB* mutant of *P. aeruginosa* that is unable to translocate T3SS effectors across membranes is capable of robust intracellular replication (123). Thus, by interfering with cytoplasmic pattern recognition receptors in macrophages, *P. aeruginosa* can survive and replicate in immune cells similarly to epithelial cells.

Taken together, current evidence suggests that *P. aeruginosa* is generally incapable of intracellular replication in immune cells in contrast to what has been reported in epithelial cells. This may be due to responses initiated through a larger repertoire of inflammasome pattern recognition receptors, which detect *P. aeruginosa* PAMPs and host cell damage in multiple ways. However, *P. aeruginosa* may also combat immune cells using defenses as a classically extracellular pathogen. Examples include inhibition of NLRC4 in macrophages by ExoT (129), inhibition of neutrophil ROS production by ExoS (130), and overall manipulation of neutrophil inflammasome responses (131). The interplay between simultaneous immune cell obstruction while neighboring epithelial cells host intracellular niches of bacteria remains to be investigated.

## INTRACELLULAR *P. AERUGINOSA IN VIVO*

A majority of the studies previously discussed have been performed *in vitro* or *in situ*, in the absence of resident immune cells *in vivo*. However, convincing evidence that invasion occurs *in vivo* comes from murine corneal infection models (16). It should be noted that the healthy cornea has fewer resident immune cells than other tissues that contain lymphatic drainage pathways and vasculature; immune responses are restricted to mitigate inflammatory damage that may alter tissue transparency (132). Although *P. aeruginosa* invasion of corneal epithelial cells occurs more efficiently *in vitro* (19), *in vivo* infection models faithfully recapitulate internalization, cytoplasmic replication, vacuolar occupancy, and blebbing (99, 133), suggesting that these phenomena are relevant to our understanding of *Pseudomonas* infection.

In line with reports of invasion of airway epithelium *in vitro* (27, 31, 37, 41, 50, 103), one pulmonary infection study in mice visualized *P. aeruginosa* isolates inside epithelial cells and macrophages (18). Beyond invasion, the capacity of *P. aeruginosa* to replicate within lung epithelial cells remains to be directly investigated *in vivo*. A recent study reported intracellular *P. aeruginosa* within lung explant specimens from CF patients undergoing transplant operations, finding them in 3 of 7 patients at varied and rare frequencies (134). In agreement with studies that have demonstrated invasion and survival *in vitro* using human bladder epithelial cells (135), a recent study from Penaranda et al. identified that *P. aeruginosa* mutants of *algR* are unable to survive within bladder epithelial cells and verified it in a mouse model of urinary tract infection (102). Here, the capacity to survive within bladder epithelial cells correlated with persistent and recurrent infections due to decreased efficacy of ciprofloxacin treatment, emphasizing potential importance of intracellular survival to the overall pathogenesis of bacteria.

Despite the physiological differences between the epithelial cells derived from various origin tissues, intracellular *P. aeruginosa* has been consistently detected at some frequency. It remains to be discerned if the intracellular lifestyle of *P. aeruginosa* occurs ubiquitously in epithelia or if tissue-specific pressures influence invasion events. Although a large body of evidence suggests that epithelial cell invasion is relevant *in vivo* for “immune privileged” tissues like the cornea, it is plausible that tissues with relatively heightened immunosurveillance like the airways and lungs may restrict *P. aeruginosa* from invading more stringently. Conversely, it is also possible that a population of *P. aeruginosa* bacteria simultaneously subvert host immune cells by inhibiting phagocytosis while persisting in epithelial cells. The myriad of proposed mechanisms of invasion discussed prior are suggestive of tissue-specific epithelial cell characteristics that may drive or restrict bacterial invasion at these different sites of infection.

## CONCLUDING REMARKS

Outstanding questions regarding invasive *P. aeruginosa* center on its relative importance in disease. Does this ability contribute to infection severity? Is invasion a component of systemic dissemination? Do intracellular bacteria resist antibiotic treatment and contribute to chronic infection? Alternatively, in what contexts might internalization of bacteria into epithelial cells benefit the host through mechanisms of clearance and suppression? Current efforts to answer these questions are limited by the fact that some virulence factors have redundant functions, and that *P. aeruginosa* uses singular virulence factors in multiple ways. Deleting or inactivating them means some functions or outcomes cannot be uncoupled. Two such examples include the duality of the T3SS apparatus and the many roles of the ADP-ribosylation activity of ExoS.

First, bacteria kill immune cells using the T3SS from an extracellular position. However, the T3SS also promotes cytoplasmic entry and replication (83). T3SS-null mutants exhibit reduced virulence presumably because they cannot avoid immune cell phagocytic killing, although they are simultaneously incapable of establishing cytoplasmic niches in epithelial cells (99). Currently, the field lacks a specific mutant that fails to occupy epithelial cell cytoplasm without *also* compromising its immune cell defenses. Exploration of genes required for metabolism or nutrient acquisition in the host cytoplasm might reveal such a tool, although it would require careful validation because this ability is unlikely to have evolved in response to mammalian cytoplasm exposure and may be needed in other contexts.

Second, ExoS ADPr activity is promiscuous (71); it executes different outcomes in different cell types (91, 136) to the benefit of keeping bacteria extracellular (137) or promoting intracellular survival (119, 122). ExoS mutants are compromised in multiple ways and exhibit reduced virulence for multiple reasons. A secondary problem is that ExoS serves a negative feedback role, repressing T3SS activation through ADP-ribosylation of an unknown host target (93). Naïve bacteria that make contact with a previously ExoS-intoxicated host cell will not normally activate type three secretion; however, they can if ExoS ADPr activity is absent. Thus, populations of ExoS-mutant bacteria hyper-secrete ExoT and ExoY due to dysregulated feedback inhibition. A tool that uncouples the impact of ExoS on host cells from T3SS regulation may not be achievable until the mechanism of contact-mediated secretion is fully understood.

The importance of investigating intracellular *P. aeruginosa* is evident, considering invasive and cytotoxic strains exhibit different characteristics during infection *in vivo* animal models (138) and clinically (139). Strains exhibiting invasive tendencies may require unique treatment for bacterial eradication; for example, benefit from topical steroid treatment also stratifies with invasive tendency in the context of corneal ulcers (139). Future studies delineating if and how *P. aeruginosa* invades epithelial cells of different tissues and how the host responds to invasion events will be critical to our overall understanding of *P. aeruginosa* infection.
